# Enterohaemorrhagic *Escherichia coli* and *Shigella dysenteriae* type 1-induced haemolytic uraemic syndrome

**DOI:** 10.1007/s00467-008-0820-3

**Published:** 2008-09-01

**Authors:** C. Mark Taylor

**Affiliations:** Department of Nephrology, Birmingham Children’s Hospital, Birmingham, B4 6NH UK

**Keywords:** Haemolytic uraemic syndrome, Aetiology, Classification, Investigation, Enterohaemorrhagic *Escherichia coli*, *Shigella dysenteriae* type-1, Shigatoxin

## Abstract

Haemolytic uraemic syndrome (HUS) can be classified according to the aetiology of the different disorders from which it is composed. The most prevalent form is that induced by shigatoxin producing *Escherichia coli* (STEC) and, in some tropical regions, by *Shigella dysenteriae* type 1. STEC cause a zoonosis, are widely distributed in nature, enter the food chain in different ways, and show regional differences. Not all STEC are human pathogens. Enterohaemorrhagic *E. coli* usually cause attachment and effacing lesions in the intestine. This is not essential, but production of a shigatoxin (Stx) is. Because Stx are encoded by a bacteriophage, this property is transferable to naïve strains. Laboratory methods have improved by identifying STEC either via the toxin or its bacteriophage. *Shigella dysenteriae* type 1 produces shigatoxin, identical to Stx-1, but also has entero-invasive properties that enterohaemorrhagic *Escherichia coli* (EHEC) do not. Shigella patients risk bacteremia and benefit from early antibiotic treatment, unlike those with EHEC.

## Introduction

Until a little over 20 years ago paediatricians were rarely able to identify the cause of haemolytic uraemic syndrome (HUS). Today, it is expected that the aetiology will be found in the majority of cases. A philosophical assumption is that each patient who meets the criteria of HUS (microangiopathic haemolytic anaemia, thrombocytopenia and renal impairment) has a distinctive disorder that is clinically recognisable. Recognition depends initially on the clinical presentation, backed up by investigations to identify environmental causes and, where necessary, inherited risk factors. Our understanding of pathogenesis lags behind that of causation. Nevertheless, there is reason to be optimistic that a modern view of the epidemiology of HUS will translate into specific treatment for specific diagnostic sub-groups in the near future.

A recently published classification of HUS based on aetiology [1] is outlined in Table 1. Two levels of diagnosis are employed. In the first, the aetiology is well established. In the second, historical descriptions and associations are used as causation remains uncertain. Increasingly, patients who would have been classified only in level 2 are becoming better investigated, sometimes retrospectively, and can be reallocated to a sub-group in level 1.

**Table 1:** An aetiological classification of HUS (see [1]). * HELLP* haemolysis, elevated liver enzymes, low platelets

Category	Characteristics
Level 1: aetiology advanced	
1.i	Infection induced
	(a) Shiga and shiga-like toxin-producing bacteria; enterohaemorrhagic *Escherichia coli*, *Shigella dysenteriae* type 1, *Citrobacter freundii*
	(b) *Streptococcus pneumoniae,* neuraminidase and T-antigen exposure
1.ii	Disorders of complement regulation
	(a) Genetic disorders of complement regulation
	(b) Acquired disorders of complement regulation, e.g. anti-factor H antibody
1.iii	von Willebrand proteinase, ADAMTS13, deficiency
	(a) Genetic disorders of ADAMTS13
	(b) Acquired ADAMTS13 deficiency; autoimmune, drug induced
1.iv	Defective cobalamin metabolism
1.v	Quinine induced
Level 2: aetiology unknown	
2.i	Human immunodeficiency virus (HIV)
2.ii	Malignancy, cancer chemotherapy and ionising radiation
2.iii	Calcineurin inhibitors and transplantation
2.iv	Pregnancy, HELLP syndrome and oral contraceptive pill
2.v	Systemic lupus erythematosus and antiphospholipid antibody syndrome
2 vi	Glomerulopathy
2.vii	Familial, not included in part 1
2.viii	Unclassified

The level 1 categories are distinct but not exclusive, so it is possible for a child to have more than one classification. This is an important point, and fits well with the concept that, often, a disease process is brought about by a combination of factors, for example a mixture of inherited risks and environmental triggers. Clinicians need to be alert to this possibility and should fully investigate any case that falls outside the locally prevalent, typical, infection-induced pattern of disease.

This review deals specifically with the aetiology of the most prevalent form of HUS, that induced by enterohaemorrhagic *Escherichia coli* (EHEC), other coliforms that produce shiga toxins, and *Shigella dysenteriae* type 1. These appear as level 1, group i. (a) in the above classification. EHEC accounts for approximately 90% of all HUS in childhood.

## Infection with shigatoxin-producing coliforms

In the early 1980s it was accepted that children who had had diarrhoea shortly before the diagnosis of HUS differed in having a relatively better outcome than those without diarrhoea [2], and the term D + HUS was subsequently adopted to describe this group. Following the seminal paper by Karmali et al. [3], it was quickly established that D + HUS was attributable to infection with shiga toxin-producing *Escherichia coli* (STEC) [4–9]. The terms shiga toxin (Stx) and verocytotoxin are equivalent. The excretion of STEC in stools can be brief, and laboratory tests for STEC infection are complex and not universally available (see below), so that the high rate of confirmation of STEC in research reports may not be mirrored in routine clinical practice. Nevertheless, in economically developed countries, the clinical features and outcome of D + HUS are broadly similar, whether or not STEC is confirmed. This suggests that, in general, cases of D + HUS have a common aetiology.

### Bacteriology

*Escherichia coli* that are capable of inducing bloody diarrhoea with or without HUS in humans are referred to as enterohaemorrhagic *E. coli* (EHEC). These organisms have various virulence factors, but the principal requirement is the ability to excrete a shiga toxin (Stx) to which humans have receptors. Shiga toxins are species restricted. Not all Stx are toxic to humans (see below), thus, not all STEC are necessarily EHEC [10]. EHEC have not acquired entero-invasive properties, and patients almost never develop septicaemia. Occasionally, STEC responsible for HUS have been recovered from the urine of patients who have not presented with gastrointestinal symptoms [11]. Whether or not these organisms have particular abilities to colonise the urinary tract has not been investigated, and septicaemia in this circumstance is described. Clearly, it is important that the urine from patients with HUS is cultured, and, if *E. coli* are found, are investigated for toxigenic properties.

EHEC usually possess additional virulence factors, such as the ability to attach to the luminal surface of host enterocytes and to cause effacement of the microvilli [12]. This property is characteristic of enteropathogenic *E. coli* (EPEC) and explains their ability to cause watery diarrhoea through loss of absorptive surface. The attaching and effacing lesion is a two-step process. The first is the ability of the *E. coli* to express the adhesin intimin on the bacterial surface. The second step is to inject intimin receptor into the host cell through a microtubular structure known as a type 3 secretion system. The intimin receptor in the host becomes orientated in the host cell membrane, permitting the *E. coli* to adhere.

Many of the proteins essential to filamentous type 3 secretion are known, and the genes that encode them occur in a pathogenicity *locus for enterocyte effacement* (LEE) [12]. These include *eaeA* the gene for intimin and *tir* the gene for the *t*ranslocated *i*ntimin *r*eceptor. EHEC seem to locate preferentially to the epithelium immediately associated with Payer’s patches, the clinical significance of which is unknown [13]. Attachment induces various signalling events in the enterocyte and cytoskeletal rearrangements, whereby the microvilli are lost and replaced by a pedestal on which the coliform is attached. There are subtle differences in signalling pathways between EHEC and EPEC [14]. It seems likely that attachment and effacement is an added virulence factor for EHEC and allows delivery of Stx in very close proximity to host enterocytes. Transcytosis of toxin across the intestinal epithelium has been demonstrated in vitro [15]. However, attachment is not an absolute requirement for EHEC, and organisms that lack this ability occasionally cause HUS.

Many EHEC also secrete calcium-dependent alpha haemolysin or possess the gene responsible for it, *ehxA* [16, 17]. Alpha haemolysin is a pore-forming toxin that induces lysis of non-human red cells and is toxic to human brain microvascular cells in vitro [18]. The association suggests that this is a virulence factor in human infection but it is not essential and its exact pathological role in haemorrhagic colitis and HUS is not known.

The *E. coli* serotypes that are associated with HUS vary in different parts of the world. In North America and North West Europe the dominant serotype is O157:H7, but other serotypes occur, either sporadically or as causes of outbreaks of enterocolitis and HUS [5, 9, 19–21]. In Southern Europe a high proportion of HUS is associated with O26 [9], and, in Australia, human infection with O157 is rare, even though Australian cattle are known to harbour it, and O111 is the dominant causative strain [22]. Several O-serotypes of EHEC are also well known as the same O-serotypes of enteropathic serotypes, for example O26, O55, O111, further illustrating the importance of the combined virulence factors of attachment and effacement. There is a steady reportage of novel serotypes, sometimes clearly traced back to animal or environmental sources (examples [23, 24])

### Toxicology

Shiga toxins have a common structure of a single A subunit linked to five B subunits (see below) [24]. Whereas the gene for shiga toxin is encoded in the chromosome of *S. dysenteriae*-1, genes for Stx1 and Stx2 are encoded by temperate bacteriophages. Bacteriophages are viruses that infect bacteria. While lytic phages are direct pathogens of the bacterial host, temperate phages can integrate with the host genome, so that the property conferred by the phage can be transmitted to subsequent generations of the bacterium. Temperate bacteriophages may be dormant for much of the life cycle of the host (prophage) but can become activated and induce lysis. A bacterium bearing a temperate phage is referred to as a lysogen.

The factors that induce lysis and regulate toxin production are not completely known. Coliforms do not appear to have a dedicated secretion system for toxin, but there is evidence that both toxin and phages are released during the phage lytic cycle Exposure of STEC to certain antibiotics appears to increase toxin release in vitro [25]. There has been concern that antibiotics use in the diarrhoeal phase of the illness might promote HUS by amplifying toxin production, but a meta-analysis, admittedly under-powered, did not confirm this [26]. EHEC can release Stx bacteriophages for uptake by other similar bacterial species, including commensal organisms, not always *E. coli*. Given that this may confer a new pathogenetic property, Stx phages are also referred to as converting phages. A clinical example that illustrates this phenomenon is that HUS has been caused by Stx2-producing *Citrobacter freundii* [27], an organism not normally associated with Stx. Moreover, an *E. coli* may have more than one phage and produce more than one toxin. A useful current review of the biology of Stx bacteriophages is provided by Allison [28].

There are two branches of the shiga toxin family. Stx1 is identical to shiga toxin, the product of *Shigella dysenteriae* type 1. Stx2 is approximately 60% homologous to Stx1, with different subtypes denoted by a suffix. For all shiga toxins the B subunit recognises and binds to a eukaryotic cell glycolipid that is expressed differently in different species. In humans this is globotriaosylceramide, Gb3, and it is expressed on renal tubular and vascular cells in kidney, brain and intestine, and in Paneth cells in the intestine, but not on intestinal epithelium [29, 30]. The receptor expression in some cells, notably glomerular endothelial cells in culture, is up-regulated by pro-inflammatory cytokines, suggesting that inflammatory events amplify toxicity [31]. Toxicity is dependent on recognition, binding and internalisation of the toxin, followed by cleavage and cytoplasmic release of the A subunit. The released A subunit is an N-glycosidase that cleaves ribosomal RNA, effectively blocking transcription (protein synthesis). Stx cytotoxicity in vitro differs in different cell types [32] and in different stages in the cell cycle. It may result in cell death in certain cell lines, but sub-lethal intoxication causes stress responses in the cell and pro-inflammatory signalling events [33]. In vascular endothelial cells this includes procoagulant effects that may be important in the pathogenesis of HUS [34].

Within a same EHEC serotype, O157 for example, one can recognise different Stx phage types that have geographical and temporal associations. EHEC responsible for HUS express Stx2 more often than Stx1 [5, 8, 35–37]. As sub-divisions of the Stx2 family become better defined, further associations are revealed, sometimes with surprises. Stx2c is positively associated with HUS disease, but Stx2d Stx2e and Stx2f. are not. Recently, a variant of Stx2d, Stx2d_(activatable)_, has been associated with HUS [38]. This toxin is modified by enzymes in intestinal mucus to increase its virulence. Moreover, EHEC-producing Stx2d_(activatable)_ seem not to require the added virulence properties of attachment and effacement. Stx2e is responsible for “oedema disease” of pigs and recognises Gb4 expressed in that species. Stx2f has been associated with birds.

A small proportion of O157 strains recovered from patients with diarrhoea and HUS do not possess Stx genes and yet, in other regards, have a closely matched genetic similarity to known toxin- and disease-producing O157 strains [39]. In some of these cases alternative Stx-producing organisms have not been found, and stools, examined by bioassay, have not contained toxin [39]. A likely explanation is that these organisms have lost the Stx gene during the course of the infection, rather than the idea that Stx-negative organisms can induce HUS [40].

### Zoonosis

STEC cause a zoonosis in which haemorrhagic colitis and HUS are the most severe expression in humans. Animals, particularly ruminants such as the cow, may be colonised by organisms that are clearly pathogenic in humans without expressing disease themselves. In cows the carriage of O157 varies according to age and feeding practices. These organisms locate to mucosal lymphoid tissue in the rectum of the cow [41]. While contamination of meat and milk products has been a major concern for food hygiene, driven by well-publicised outbreaks involving undercooked ground beef, this is probably not the most common route for human exposure. STEC shed on to pastureland survive over a wide range of temperatures and pHs and resist composting. Because they persist in soil, they can readily contaminate surface water. Salads and vegetables may, therefore, become contaminated, as well as domestic water supplies. However, in small children, an important risk appears to be direct contact with animals, for example from visits to farms [42]. Unsurprisingly, in industrialised countries, there is a seasonal pattern to the incidence of HUS, being greater in summer than in winter. This probably reflects increased exposure to countryside, animals and fresh uncooked foods. Bathing in contaminated water is an additional risk [24].

Humans acquire antibodies to Stx2 during childhood and teenage years, so that approximately half of adults have antibodies detectable by western blotting [36]. Stx antibodies wane in old age. Family studies have found antibodies in close contacts of children with haemorrhagic colitis and HUS, particularly their carers. Some of these will have had minor gastrointestinal symptoms, and others may be asymptomatic, which suggests that sub-clinical infection might be quite common [43]. A high proportion of abattoir workers have been shown to excrete STEC, although not necessarily EHEC [44]. This raises the possibility that contact with STEC that produce Stx2 but lack the necessary additional virulence factors needed for a human pathogen might be immunogenic. Experimentally, anti-Stx antibodies are protective in several models of EHEC infection, and it is a reasonable hypothesis that adults are, to some extent, protected by acquiring anti-Stx2, and that the high incidence of HUS in pre-school children reflects immunological naivety.

### Incidence

The greatest incidence of EHEC-induced HUS in Europe and North America is in children aged 1–5 years, although it can occur after that age, whereas, in Argentina, the age of onset is lower, between 6 months and 4 years. The onset of HUS much before 6 months of age would raise concern that EHEC was unlikely to be the only cause. The incidence also differs between regions, generally being higher in cooler temperate regions. For example, the incidence in Scotland (3.4 per 10^5^ children < 5 years of age) is greater than in England (1.54 per 10^5^ children < 5 years of age) [45]. The incidence in England and France is similar and greater than in Italy [9]. While the diagnosis of EHEC infection has increased over time, perhaps reflecting better laboratory techniques, the number of children presenting with HUS has remained stable over the past 20 years in economically developed states, and the mortality has reduced. However, fluctuations in incidence may occur, with local epidemics sometimes linked to a common source of infection. In such outbreaks it has been estimated that one in ten exposed to the infection develops symptoms of colicky abdominal pain and diarrhoea, and 15% of children with diarrhoea or bloody diarrhoea will develop HUS. Outbreaks may be biphasic, a second wave occurring 2 weeks later from person-to-person transmission. The time from exposure to onset of diarrhoea is usually less than a week, mostly 3–4 days, and the mean interval between onset of diarrhoea and disclosure of HUS is 4 days, range 1–10 days.

### Laboratory investigation

In regions where O157 is the predominate EHEC it has been common and economic practice to culture stools on sorbitol MacConkey agar enriched with tellurite. The latter promotes the growth of O157. Because this serotype is usually unable to ferment sorbitol, colonies can be inspected, picked, and then tested specifically for O157 by agglutination or enzyme immunoassay (EIA). This approach will miss other serotypes of EHEC and any O157 that is capable of fermenting sorbitol. It will also identify Stx-negative strains of O157.

Given that Stx production is an essential feature of EHEC, and central to the epidemiology, it is logical to investigate directly for this property rather than rely on identifying O-serotypes that may or may not be toxin producers [46]. Historically, toxin identification was laborious and expensive. Stools were filtered to obtain free toxin that was tested on verocell cultures, confirmation being sought by neutralisation of the toxin with specific antibodies.

Sensitive immunoassays to detect toxin, such as BioStar, are now commercially available and can be applied directly to stool samples or cultures [47]. The sensitivity and specificity of this rapid diagnostic test suggest that it will become the screening test of choice. Polymerase chain reaction and DNA hybridization can also be used to amplify and detect Stx genes. This in itself does not identify the organism, but colonies with these properties can be further identified for serotype and other markers.

Genetic profiling of EHEC is valuable in research and in identifying emerging pathogenic strains. Another established technique is lytic phage typing. This, for example, was used to show the emergence of a new strain of O157 in the United Kingdom in the 1990s [45]

## Infection with *Shigella dysenteriae* type 1

HUS is a well-recognised complication of *Shigella dysenteriae* type 1 infection. Many of the features of the syndrome resemble EHEC-induced HUS. The age range is wider, the median age of presentation being approximately 3 years, and the median time from the onset of diarrhoea to the presentation of HUS is 7 days, compared to 4 for most EHEC infections [48].

*Shigella dysenteriae* can be entero-invasive, while EHEC typically are not. Therefore, unlike EHEC infection, early and appropriate antibiotic treatment is indicated and appears to reduce the incidence of HUS [49]. Shiga toxin is implicated in the pathogenesis. In some laboratory models of HUS a combination of a ribotoxin and lipopolysaccharide is more likely to induce glomerular thrombosis than is toxin alone [50, 51]. Children with *Shigella dysenteriae*-induced HUS are exposed to bacterial lipopolysaccharide because of the entero-invasive nature of the organism, and this added stimulus is likely to be pathogenic [52]. The neutrophilia at onset is typically greater than that with EHEC but gives a similar prediction for the development and severity of HUS [53, 54]. In some patients disseminated intravascular coagulation leads to consumption of coagulation factors, a very rare event in EHEC-induced HUS. It is customary to exclude disseminated intravascular coagulation with consumption of coagulation factors, such as that seen in sepsis and multi-organ failure, from within the term HUS. However, *Shigella dysenteriae* and invasive *Streptococcus pneumoniae* infections that cause HUS elude that restriction. It is likely that coagulation tests in the microangiopathic haemolytic anaemia of Shigella-induced (and pneumococcus-induced) HUS at least begins with normal or activated coagulation.

There is the general impression that HUS complicating *Shigella dysenteriae* is more severe, but the condition mostly occurs in developing countries in tropical regions, where children may have co-morbidities and poor access to health care. Catastrophic dehydration, hyponatraemia and central nervous system complications may, in part, reflect this. In epidemics in sub-Saharan Africa mortality rates of 17% and 43% are described [48, 55], whereas, in an outbreak in France, all five affected children recovered with normal renal function [56].

## Questions

(Answers appear after the reference list)

- Which of the following are necessary for *Escherichia coli* to cause HUS?
- Intimin and translocated intimin receptor
- Shigatoxin
- Entero-invasive properties
- Minimum infective oral dose > 10^9^ organisms
- Specific O-serotype (e.g. O157)
- Which of the following scenarios suggest a cause other than EHEC infection?
- Two months old, with HUS preceded by diarrhoea
- A 13-year-old girl with HUS who had bloody diarrhoea a week ago while on a camping holiday
- Infant with HUS complicating pneumonia and empyema
- A 2 year old with D + HUS whose stools exhibited repeatedly negative findings for *E. coli* O157, (sorbitol MacConkey culture and O157 antibody agglutination test)
- A teenager awaiting cadaveric transplantation after a single episode of D + HUS. His brother died of HUS 10 years ago aged 8 years.
- Which of the following is true?
- Shigatoxin2 (Stx2) is encoded in the chromosome of Shigella dysenteriae type 1
- Shigatoxin1 (Stx1) is more often associated with HUS than is Stx2
- Shigatoxins do not elicit an antibody response in humans
- All Stx-producing *E. coli* are pathogenic in humans
- Stx access mammalian cells through specific receptors, Gb3 in humans
- In investigation of an outbreak of D + HUS, which of the following tests provides the best chance of identifying a new strain of enterohaemorrhagic *E. coli*?
- Selective culture of stool sample on sorbitol MacConkey agar
- Immuno-magnetic bead separation of stool sample
- Blood culture (aerobic)
- Polymerase chain reaction (PCR) amplification and DNA hybridization of Stx phages
- Paired serological examination for O-serotypes
- Which of the following are false?
- Enterohaemorrhagic *E. coli* may colonise the urinary tract
- Stx2e is associated with porcine not human toxicity
- Antibiotics are contraindicated in *Shigella dysenteriae*-induced HUS
- Antibodies against Stx2 are likely to protect against HUS
- *Shigella dysenteriae* type 1 is entero-invasive
